# Supplementary material for: Multidrug-resistant and extended-spectrum beta-lactamase-producing Enterobacteriaceae isolated from chicken droppings in poultry farms at Gondar City, Northwest Ethiopia
Source: PLoS One. 2023 Jun 9;18(6):e0287043. doi: 10.1371/journal.pone.0287043 (PMC10256222; doi:10.1371/journal.pone.0287043)
Supplement: S1 File — (DOCX) [file pone.0287043.s001.docx]

**Questionnaire English version**

Questionnaires to collect data on factors to MDR and ESBL-producing Enterobacteriaceae isolated from chicken droppings from poultry farms in Gondar city, Northwest Ethiopia.

Identification

Questionnaire number/Code________________ Name of interviewer __________

Date of interview / 2022

| Section I: General information on poultry farm characteristics | | |
| --- | --- | --- |
| No. | Questions | Classification and category code |
| 1.1 | Age of chickens? | ……………………………. |
| 1.2 | How old your farm age (years)? | …………………………….. |
| 1.3 | How many chickens are found in your farm currently? | ………………………. |
| 1.4 | What type of your commercial chicken? | 1. Layer 2. Broiler 3. Dual purpose 4. One day old |
| 1.5 | What is the type of housing you use for your chicken? | 1. Deep litter system 2. Traditional housing |
| 1.6 | Do you isolate and separate diseased chickens timely? | 1. Yes 2. No |
| 1.7 | By how much time do you clean chicken droppings? | --------------------- |
| 1.8 | How you dispose waste/litter from your farm? | 1. Send to field/anywhere 2. Compost |
| 1.9 | Does professional farm training is given? | 1. Yes 2. No |
| 1.10 | Do owners and chicken caregiver profession-related poultry production? | 1. Yes 2. No |
| 1.11 | What is the source of feed for your chicken? | 1. Commercially formulated  2. Locally formulated  3. Both |
| 1.12 | What is the source of water are you using for chicken on your farm? | 1. Well water  2. Pipe water |
| 1.13 | Does chicken feeds contact with their droppings? | Yes, 2. No |

**Laboratory activity**

**Culture of media preparation**

**Selenite F broth**

Selenite F broth is used as a selective enrichment for the cultivation of *Salmonella* spp. and some species of *Shigella*.

Preparation of Selenite F broth

1. Prepare Selenite F broth according to the manufacturer's instructions.
2. Add 4.0 gm sodium biselenite powder to distilled water.
3. Add the remaining 19.0 gm of Selenite F broth powder to the above solution and bring the volume to 1000 litres.
4. Gently heat and bring to boiling.
5. Dispense into sterile test tubes, at least at a depth of 5 ml.
6. Sterilize in a boiling water bath at 100°C for 10 minutes.
7. **Do not autoclave.**
8. Cool to room temperature before use.

**Buffered peptone water**

1. Prepare BPW according to the manufacturer's instructions
2. Preparation dissolves the dehydrated medium in the water by heating if necessary.
3. Transfer to a test tube
4. Autoclave at 121^o^C for 15 min.
5. Place the tubes in sterile plastic bags and store them at 4-8ºC until use.

**MacConkey agar**

MacConkey agar is used for the isolation of gram-negative enteric bacteria and differentiation of lactose fermenting, from lactose non-fermenting bacteria.

**Procedure**

1. Prepare MacConkey agar according to the manufacturer's instructions.
2. Sterilize the medium by autoclaving at 121°C for 15 minutes.
3. Cool to 50°C in a water bath.
4. Dispense 20 ml into 15x100 mm Petri dishes. Allow the media to solidify and condensation to dry.
5. Place the plates in sterile plastic bags and store them at 4-8ºC until use.

**Xylose lysine deoxycholate agar**

It is a selective differential medium for the isolation of Gram-negative enteric pathogens from fecal samples and other clinical material and is also used for microbiological testing of foods, water and dairy products and it is especially suitable for the isolation of *Shigella* and *Salmonella* species.

**Procedure**

1. Prepare XLD according to the manufacturer's instructions by suspending 55 grams of the dehydrated medium in 1000 ml distilled water.
2. Heat with frequent agitation until the medium boils

*Note:* ***do not autoclave.***

1. Transfer immediately to a water bath at 50°C.
2. Dispense 20 ml into 100 mm Petri dishes. Allow the media to solidify and condensation to dry.
3. Place the plates in sterile plastic bags and store at 4-8ºC until use

**Mueller-Hinton agar**

**Procedure**

- 1. Follow the manufacturer's instructions to prepare MHA from a commercially available dehydrated base.
  2. After autoclaving, cool the agar in a 45°C to 50°C water bath.
  3. Pour agar into Petri dishes on a level pouring surface.
- Measure 60-70 ml medium per plate into 15x150 mm plates or measure 25-30 ml per plate into 15x100 mm plates to give a uniform depth of approximately 4 mm.
  1. Allow the media to solidify and condensation to dry.
  2. The pH of MHA should be 7.2-7.4.
  3. Place the plates in sterile plastic bags and store them at 4ºC until use.

**Gram’s stain preparation and examinations**

Gram staining is the type of differential staining which is common, important, and most used. This method differentiates the bacteria into gram-positive and gram-negative.

**Reagents used and materials required**

- Crystal violet, the primary stain
- Gram’s iodine, the mordant
- A decolourizer made of acetone and alcohol (95%)
- Safranin, the counterstain
- Inoculating loop
- Bunsen burner
- Bibulous paper
- Microscope
- Immersion oil
- Distilled water
- 18 to 24-hour cultures of the organism

**The procedure of Gram staining**

1. Labeling the slides clearly with the code number
2. With a sterile cooled loop, place a loopful of the culture on the slide.
3. Spread using a circular motion of the inoculating loop.
4. Air dry and heat fix
5. Place the slide with a heat-fixed smear on the staining tray.
6. Gently flood smear with crystal violet and let stand for 1 minute.
7. Tilt the slide slightly and gently rinse with tap water using a wash bottle.
8. Gently flood the smear with Gram’s iodine and let stand for 1 minute.
9. Tilt the slide slightly and gently rinse with tap water using a wash bottle.
10. The smear will appear as a purple circle on the slide.
11. Decolorize using acetone alcohol.
12. Tilt the slide slightly and apply the alcohol drop by drop for 30 seconds until the alcohol runs almost clear.
13. Immediately rinse with water.
14. Gently flood with safranin to counter-stain and let stand for 45 seconds.
15. Tilt the slide slightly and gently rinse with tap water using a wash bottle.
16. Blot dry the slide with bibulous paper.
17. Examine the smear using a light microscope under oil immersion.
18. And continue the next procedure if the result may

Gram-negative: **pink colour, rod in shape**

**Biochemical test**

For the identification of Enterobacteriaceae*,* we have used the following biochemical tests medium.

- Triple Sugar Iron: which has multi-purposes, helps to see fermentation of carbohydrates, production of gas and H_2_S
- Indole: helps to see the production of tryptophanase enzyme which breaks tryptophan and then produces indole
- Urea: shows the ability of the isolates to hydrolyze urea by producing the enzyme urease
- Citrate: it is essential to see the bacterial ability to utilize it as the sole carbon source
- Motility: helps to see the ability of an organism to move by itself using propeller-like flagella
- Lysine decarboxylase: lysine gets decarboxylated when Enterobacteriaceae isolates produce lysine decarboxylase enzyme

**Procedure**

1. Prepare a suspension of the test bacterium by normal saline with compared to 0.5 McFarland standard.
2. A loop full of the bacterial suspension is inoculated in sulfur indole motility, urea, triple sugar iron, citrate, and lysine decarboxylase.
3. Incubate at 37^O^c + 2^0^C for 18-24 hours
4. Look for the production of indole by adding Kovacs reagent in tryptophan broth
5. Look for colour change in the other test medium (turbidity for motility) of the medium
6. Identify the test organism by following the standard biochemical test result chart for Enterobacteriaceae.

Table; Biochemical characteristics of isolated bacteria

|  | Standard biochemical test for Enterobacteriaceae | | | | | | | | |
| --- | --- | --- | --- | --- | --- | --- | --- | --- | --- |
| Bacterial isolate | TSI | | | | Citrate test | Urease test | SIM | | LDC |
|  | Slant | Butt | H2S | Gas |  |  | Indole | Motility |  |
| *E. coli* | A | A | -VE | +VE | -VE | -VE | +VE | +VE | +VE |
| *K. pneumoniae* | A | A | -VE | +VE | +VE | +VE | -VE | -VE | +VE |
| *P. mirabilis* | K | A | +VE | +VE | +VE | +VE | -VE | +VE | +VE |
| *Salmonella* species | K | A | +VE | +/-VE | +VE | -VE | -VE | +VE | +VE |
| *E. cloacae* | A | A | -VE | +VE | +VE | -VE | -VE | +VE | -VE |

Key: A= Acid, K=Alkaline, TSI= Triple sugar iron, SIM=Sulphur indole motility, LDC= Lysin decarboxylase, H2S= Hydrogen sulfide, +VE=Positives, -VE=Negative

**Antimicrobial Susceptibility testing**

**Method**: Kirby-Bauer disk diffusion susceptibility testing technique

**Procedure:**

1. Using a sterile wire loop prepare suspensions in sterile normal saline with equal to 0.5 McFarland standard.
2. Using a sterile swab, inoculate the MHA plate. Streak the swab evenly over the surface of the medium in three directions, rotating the plate for approximately 60^o^ to ensure even distribution.
3. With the petri dish lid in place, allow 3-5 minutes (*no longer than 15 minutes*) for the surface of the agar to dry.
4. Using sterile forceps placed the appropriate antimicrobial disks, evenly distributed on the inoculated plate. ***Note****:* The disks should be about 15 mm from the edge of the plate and no closer than about 24 mm from disk to disk. No more than 6 disks and 12 disks should be applied on the 90 mm dish and, 150 mm dish, respectively. Each disk should be lightly pressed down to ensure its contact with the agar.
5. Within 30 minutes of applying the disks, invert the plate and incubate aerobically at 35^o^C for 16-18 hours.
6. After overnight incubation, examine the control and test plates to ensure the growth is confluent or near confluent. Using a ruler on the underside of the plate measures the diameter of each zone of inhibition in mm.
7. Interpretation of zone sizes based on CLSI criteria and reporting the organism as resistant, intermediate, and susceptible

**Table: AST interpretive chart for Enterobacteriaceae**

| S.No | Antibiotic agents | Disk content | CLSI 2021, Interpretive category in mm | | |
| --- | --- | --- | --- | --- | --- |
|  |  |  | Sensitive | Intermediate | Resistance |
| 1 | Ampicillin | 10 µg | **≥ 17** | **14-16** | **≤ 13** |
| 2 | Gentamicin | 10µg | **≥ 15** | **13-14** | **≤ 12** |
| 3 | Tetracycline | 30 µg | **≥ 15** | **12-14** | **≤ 11** |
| 4 | Chloramphenicol | 30 µg | **≥ 18** | **13-17** | **≤ 12** |
| 5 | Nalidixic acid | 30 µg | **≥ 19** | **14-18** | **≤13** |
| 6 | Ciprofloxacin | 5 µg | **≥ 26** | **22-25** | **≤ 21** |
| 7 | Cefoxitin | 30 µg | **≥ 18** | **15-17** | **≤ 14** |
| 8 | Meropenem | 10 µg | **≥ 23** | **20-22** | **≤ 19** |
| 9 | **Ceftriaxone** | 30 µg | **≥ 23** | **20-22** | **≤ 19** |
| 10 | **Ceftazidime** | 30 µg | **≥ 21** | **18-20** | **≤ 17** |
| 11 | **Cefotaxime** | 30 µg | **≥ 26** | **23-25** | **≤ 22** |
| 12 | Trimethoprim-sulfamethoxazole | 1.25/23.75 µg | **≥ 16** | **11-15** | **≤ 10** |

**AST registration chart**

Code ……….………………………. **Type of isolate**………………………………………….

**AST result**: Ampicillin………………...Gentamicin………………Tetracycline ………………

Chloramphenicol …………………… Nalidixic acid ………………Ciprofloxacin………………

Cefoxitin …………................. Meropenem ………………… Ceftriaxone ………………………

Cefotaxime ……………………. Ceftazidime ………………………………Trimethoprim-sulfamethoxazole…………………………**ESBL- Result**………………………………

**ESBL detection**

**Method:** Combined disk diffusion technique

**Required materials:** suspected isolate, MHA, disks containing cephalosporin alone (cefotaxime, ceftazidime) and in combination with clavulanic acid and control strains to test the effectiveness of the disks.

**Test procedure**

1. Using a fresh, pure culture prepares a suspension of the test organism equal to 0.5 McFarland standard.
2. Using a sterile cotton swab spread the adjusted suspension over the entire area of MHA plate.
3. Apply the disks onto the inoculated plate, ensuring sufficient space between individual disks to allow for the proper measurement of inhibition zones.
4. Incubate at 35±2°C for 18-24 hours.
5. At the end of the incubation period, the inhibition zone around the cephalosporin disk combined with clavulanic acid is compared with the zone around the disk with the cephalosporin alone. The test is positive if the inhibition zone diameter is ≥ 5 mm larger with clavulanic acid than without.

**ESBL screening and confirmatory test interpretive**

| ESBL Screening test will pass | | | If Inhibition zone | | ESBL Confirming | | Interpretation |
| --- | --- | --- | --- | --- | --- | --- | --- |
| Ceftazidime | 30 µg | | **≤ 22mm** | | Ceftazidime 30 µg  Ceftazidime-clavulanate 30 /10 µg | | **A ≥ 5mm** increase in the zone for antimicrobial agent tested in **combination with clavulanate vs tested alone**, ESBL positive, Unless ESBL Negative |
| Cefotaxime | 30 µg | | **≤ 27mm** | | Cefotaxime 30 µg  Cefotaxime-clavulanate 30 /10 µg | |  |
| Ceftriaxone | 30 µg | | **≤ 25mm** | |  | |  |
| **Positive quality control for ESBL: *K. pneumonia* ATCC 700603, acceptable QC range** | | | | | | | |
| Ceftazidime | | **10-18mm** | | **≥ 5mm** increase in zone diameter of Ceftazidime-clavulanate vs Ceftazidime alone | | | |
| Cefotaxime | | **17-25mm** | | **≥ 3mm** increase in zone diameter of Cefotaxime-clavulanate vs Cefotaxime alone | | | |
| **Negative quality control for ESBL: *E. coli* ATCC 25922, acceptable QC range** | | | | | | | |
| Ceftazidime | **25-30 mm** | | | | | **≤ 2mm** increase in zone diameter for antimicrobial agent tested in combination with clavulanate vs tested alone | |
| Cefotaxime | **29-35 mm** | | | | |  |  |


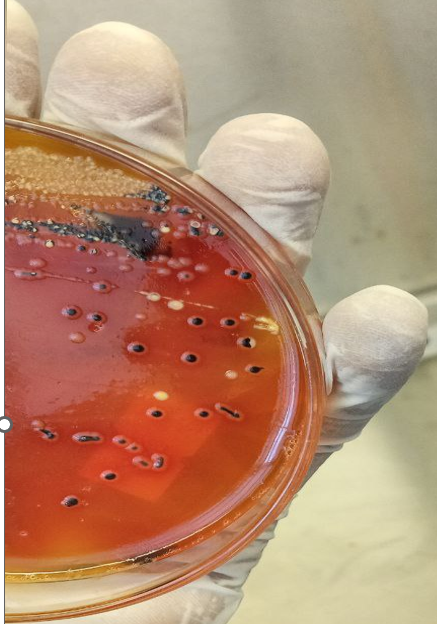
**Photos of laboratory activity**


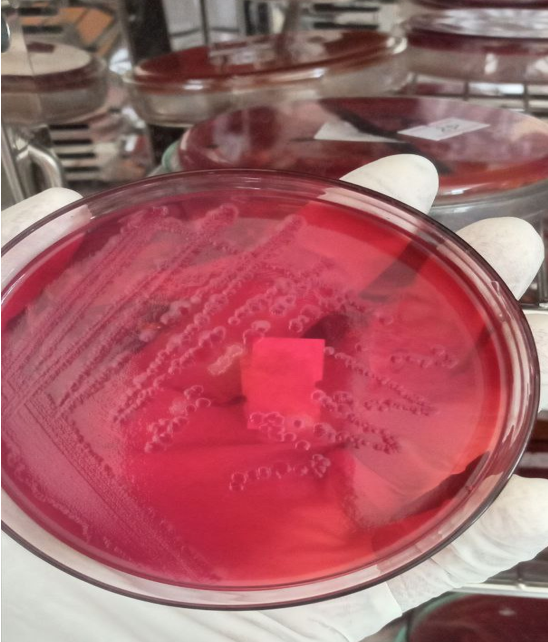


Fig Lactose fermenter colony on MacConkey and Salmonella suspected colonies on XLD agar respectively.


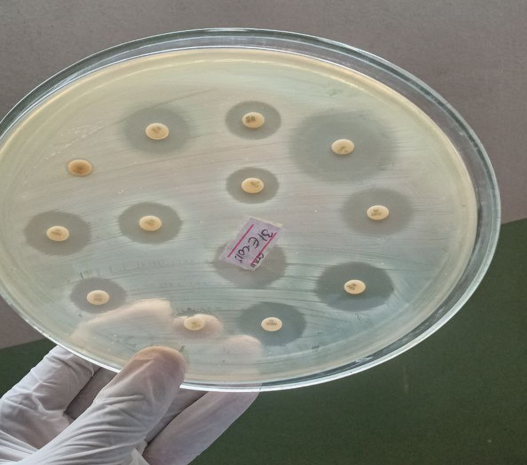


Fig AST result to *E. coli* on MHA

**Photos of laboratory activity on ESBL-result**


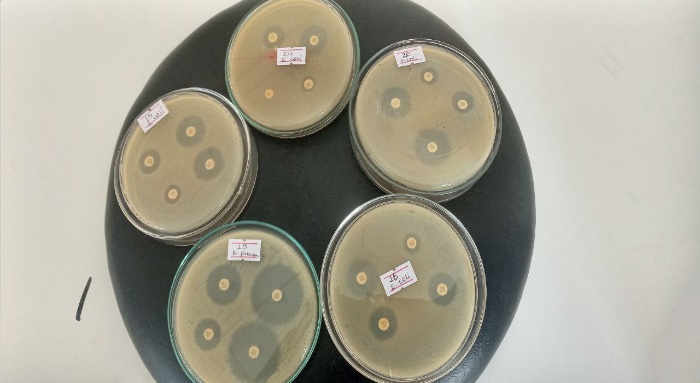


Fig ESBL producing *E. coli* and *K. pneumonia*e isolates


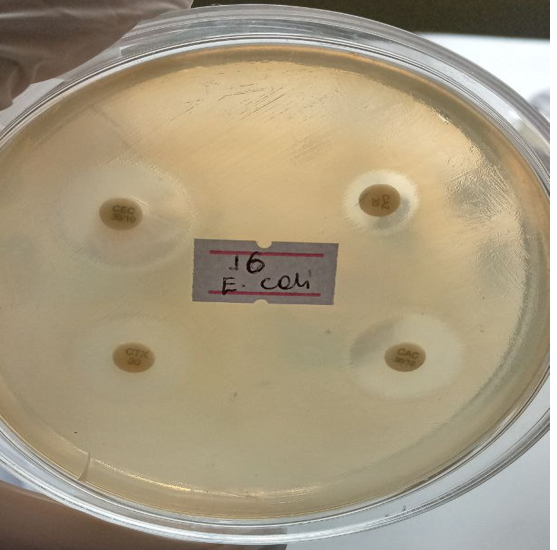


CAC (30/10 µg) =18 mm

CEC (30/10 µg) =22 mm

CTX (30 µg) =17

CAZ (30µg) =11 mm

Fig: Example of ESBL-producing *E. col*
