# Supplementary material for: Multidrug-resistant and extended-spectrum beta-lactamase-producing Enterobacteriaceae isolated from chicken droppings in poultry farms at Gondar City, Northwest Ethiopia
Source: PLoS One. 2023 Jun 9;18(6):e0287043. doi: 10.1371/journal.pone.0287043 (PMC10256222; doi:10.1371/journal.pone.0287043)
Supplement: Attachment — Submitted filename: Response to Reviewers.docx [file pone.0287043.s002.docx]

**Response to Reviewers**

To journal editors and reviewers:

**Authors:** We appreciate for spending your precious time and forwarding your valuable comments, which have significantly improved our application. We are also grateful for this positive feedback. Please see below, bold, for a point-by-point response to the reviewers. All page numbers refer to the revised manuscript file with tracked changes. All modifications in the manuscript have been highlighted in red.

**Editor:** Please ensure that your manuscript meets PLOS ONE's style requirements, including those for file naming.

**Authors: Thank you for your positive feedback. We believed that we are following PLOS ONE’s style requirements.**

**Editor:** We note that you have stated that you will provide repository information for your data at acceptance. Should your manuscript be accepted for publication, we will hold it until you provide the relevant accession numbers or DOIs necessary to access your data. If you wish to make changes to your Data Availability statement, please describe these changes in your cover letter and we will update your Data Availability statement to reflect the information you provide.

**Authors: Thank you. Please update it**

**Reviewer #1**

**Authors: We appreciate you taking the time to thoroughly review this manuscript and provide us with constructive feedback, which we believe improved the overall quality of our paper. We made every effort to address all your concerns. We've copied your comments and responses below to make things easier for you.**

**Reviewer #1**: There are many places in the manuscript written wrongly used words, phrases and sentences. English correction is needed for sentence to sentence.

**Authors: Thank you for your input. We attempted to review the entire document and make any necessary changes, and We modified the English corrections by native English speakers.**

**Reviewer #1**: The theme of the abstract is not highlighted/reflected to emphasize the title. Please modify the abstract with noting a brief primary objective.

**Authors**: **Thank you for your comment. We tried to modify the abstract section**

**Reviewer #1**: The introduction section is very weak and uninformative. Please strengthen the introduction with relevant citations. There is so much languages problem. Please modify it by Native English Speaker. What is the significance of the study? How is the study different from others? Please note it down and rectify it accordingly.

**Authors**: **We appreciate you bringing these concerns to our attention.** **We tried to update the introduction section to make it stronger and more understandable. Even though there are few published reports in Ethiopia focused on the antibiotic resistance pattern of Enterobacteriaceae, our study included multiple poultry farms and different types of commercial chickens such as layer, broiler, and day-old chickens. In addition to the antibiotic resistance pattern, the ESBL status of Enterobacteriaceae was also assessed. We amended the significance of the study and explain how our study is different from others.** (**Please refer to the introduction section of the revised manuscript).
Reviewer #1**: Results and discussion portion is haphazardly written and difficult to correlate with each other. Please modify the discussion in a systemic manner with relevant references. Please modify the discussion by strengthening the results. There is also lacuna of the representation of the discussion in the present endeavor. Please modify the discussion with proper justification and analysis by strengthening the novelty of the work.

**Authors:** **We appreciate your feedback and suggestions. We tried to address all your concerns in the revised manuscript**. (**Please refer to the discussion section of lines #344-364 in the revised manuscript).**

**Reviewer** **#2**

**Authors**: **We appreciate you taking the time to thoroughly review this manuscript and provide us with constructive feedback, which we believe improved the overall quality of our paper. We made every effort to address all your concerns. To make things easier for you, we've copied your comments below, along with our responses.**

**Abstract**

**Reviewer #2:** While the author aimed to Enterobacteriaceae, they have mentioned only enrichment and isolation of Salmonella. They said the isolation of E. coli, Salmonella and P. mirabilis. But, just next to that they mentioned 9.1% of K. pneumonia as ESBL!! Strange, why didn’t they mention K. pneumonia earlier?

**Authors**: **Thank you for your input. We tried to mention important isolates like K. pneumonia in the** **abstract section and make any necessary changes. (Please refer to line # 43 in the abstract section of the revised manuscript). We didn’t mention the prevalence of *E. cloacae* due to considering the prevalence of *E. cloacae* was low. we mentioned only the enrichment media for *Salmonella* because *Salmonella* spp. are fastidious pathogen which requires enrichment media.**

**Introduction**
**Reviewer #2:** The importance is nicely described with necessary references; however, this could be further improved with information on ESBL. Few typos mistake.

**Authors**: **Thank you for your suggestions. We attempted to revise the introduction and include some information that we believe strengthens the manuscript (Please refer to the introduction section of the revised manuscript). We tried to amend topographic errors**.

**Materials and methods**
**Reviewer #2:** Although they targeted Enterobacteriaceae, isolation was performed on MacConkey and XLD agar which limits the isolation of other enterobacteria and increases the chance of missing bacteria that does not grow on those media properly. So, my suggestion is, instead of saying Enterobacteriaceae, they could mention specific bacteria like E. coli and Salmonella

**Authors**: **Thank you for pointing this out. MacConkey and XLD agar are the two most common media used by many researchers targeting Enterobacteriaceae. We know that other media in addition to those media can better recover all Enterobacteriaceae. We couldn’t use additional media because of the limited capacity of our microbiology laboratory. We included this information in the** **limitation part**. **(Please refer to** **the** **limitation section of the revised manuscript** **line # 366–368).**

**Reviewer #2:** The authors didn’t mention, how they calculated sample size, i.e. no sample size calculation was performed!!

**Authors**: **Thank you for your comment. We didn’t calculate the sample this study is a survey study it included all poultry farms found in the study area.**

**Reviewer #2:** Line 158: 5%???

**Authors: Thank you for your positive feedback. We tried to modify it in an understandable way. in the revised manuscript (refer to line # 177) and 5% which was found on line 158 indicates when we prepared culture media, 5% of prepared culture media incubated at 35-37 °C overnight without inoculation and was evaluated for possible contamination. If there is growth, we will discard all prepared media. For example, if we prepared culture media into 100 Petri dishes or test tubes, 20 Petri dishes or test tubes will incubate at 35-37 °C overnight to check the sterility of the procedure.**

**Results**
**Reviewer #2:** Nicely presented with easy-going Tables. In Prevalence, they didn’t mention about *K. pneumonia*, although its prevalence was not that low. I think it worth to mention in the prevalence section. In addition, they also isolated *E. cloacae*, but no mention in the prevalence or isolation section (Table 4) But, in Antibiotic resistance *K. pneumonia* suddenly came (Table 3)!!

**Authors**: **Thank you for bringing this issue to our intention. We considered the prevalence of *K. pneumonia* and *E. cloacae* to be low. We didn’t mention these bacteria in the text part because we have already presented them in Figure 1. But now we tried to mention *K. pneumonia* in the prevalence section in a text**. **(Please refer to line # 223 in** **the revised manuscript).**

**Discussion**
**Reviewer #2:** The authors have discussed the findings nicely with evidences from different part of the world. However, the authors have identified several general characteristics significantly associated with the occurrence of ESBL, but they didn’t discussion anything about them.
The discussion feels very monotonous. It could be improved with suggestion from a person with good English writing skill.

**Authors**: **Thank you for your input. We tried to address all your concerns in the revised manuscript**. (**Please refer to lines # 343 – 364 in the revised manuscript).**

**Conclusion and recommendations**
**Reviewer #2:** Waste disposal was not significantly associated with occurrence of ESBL according to their findings (Table 1). Rather, some critical factors were identified, which could be highlighted in the conclusion section.

**Authors: Thank you for reflecting on your concern. We amended and made the necessary changes. Please refer to lines # 374 – 375 in the revised manuscript).**

**Authors**: **Finally, we would like to say thank you for reviewing our work and making insightful suggestions and comments that helped to strengthen our manuscript. We revised the manuscript as necessary.**
